# Letter from the Editor in Chief

**DOI:** 10.19102/icrm.2024.15036

**Published:** 2024-03-15

**Authors:** Moussa Mansour


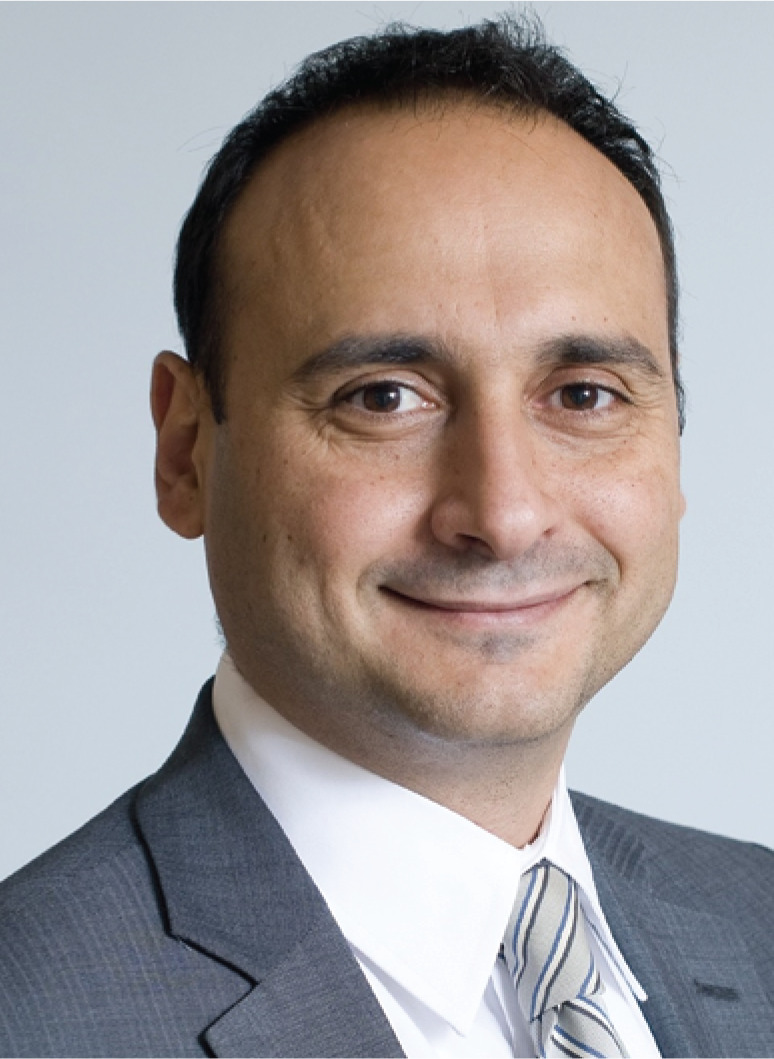


Dear readers,

This issue of *The Journal of Innovations in Cardiac Rhythm Management* contains many interesting articles. I would like to highlight a case report by Kerley et al. titled “Refractory Inappropriate Sinus Tachycardia Treated with Pulsed-field Ablation of the Sinus Node: A Breath of Fresh Air,” which was a finalist in the EP Fellows Summit 2023 case competition.^1^ In it, the authors describe the case of a young patient with incessant inappropriate sinus node tachycardia refractory to conventional therapies. Because of the proximity of the target area to the phrenic nerve, modification of the sinus node was successfully performed with pulsed-field ablation (PFA) using the pentaspline catheter instead of radiofrequency energy. This case is important because it highlights the safety of PFA and its effect on the phrenic nerve.

PFA is a largely non-thermal energy approach that involves the use of microsecond-scale, high-voltage electric fields to cause irreversible electroporation and destabilization of cell membranes, a process that culminates in selective cellular necrosis.^2^ Over the past several years, numerous pre-clinical studies have demonstrated the selectivity of PFA for cardiac tissue and its lack of effect on collateral structures such as the phrenic nerve.^3^ The findings of pre-clinical studies were corroborated in investigational studies and real-world experiences, which demonstrated the safety of PFA. The latest data came from the Multi-national Survey on the Safety of the Post-approval Clinical Use of Pulsed Field Ablation in 17,000+ Patients (MANIFEST 17K) trial presented by Dr. Vivek Reddy at the AF Symposium in February 2024. The study included 17,642 patients from 106 European centers and showed no persistent phrenic nerve paralysis. There were, however, rare cases of transient phrenic nerve injury in 0.06% of the patients.

The data from MANIFEST 17K and other studies demonstrate that PFA is safer than thermal ablation with regard to phrenic nerve injury. However, it is important to remember that these studies included patients undergoing pulmonary vein isolation. As a result, caution should be exercised when extending the findings to ablation in the lateral right atrium, where energy delivery could be closer to the phrenic nerve. In addition, cases of transient phrenic nerve paresis have been described, indicating that injury of the phrenic nerve can occur with PFA, albeit at a significantly lower degree than with thermal ablation and with the ability to be reversed. In the case report by Kerley et al., the authors should be praised for their caution, as evidenced by using a stepwise graded approach with PFA in a patient who failed all other forms of conventional therapies.

PFA is now widely available, and its use has been gradually replacing thermal ablation. It is safer, faster, easier to use, and probably more effective than thermal ablation for the treatment of atrial fibrillation. However, like using any new technology, caution should be exercised when using it outside the areas tested in clinical trials.

I hope that you enjoy reading the remaining articles in this issue of *The Journal of Innovations in Cardiac Rhythm Management*.


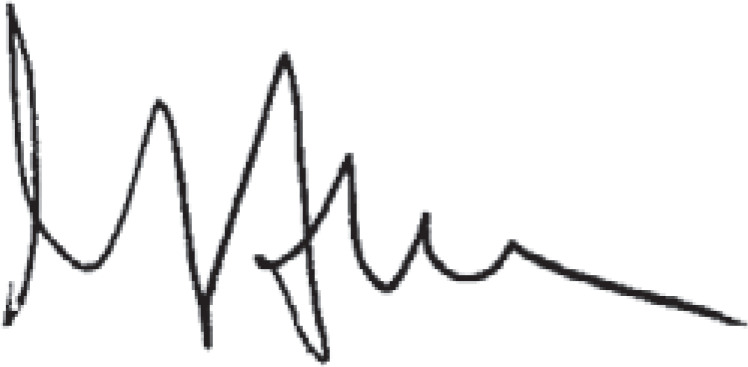


Best regards,

Moussa Mansour, md, fhrs, facc

Editor in Chief

*The Journal of Innovations in Cardiac Rhythm Management*

MMansour@InnovationsInCRM.com

Director, Atrial Fibrillation Program

Jeremy Ruskin and Dan Starks Endowed Chair in Cardiology

Massachusetts General Hospital

Boston, MA 02114
